# Health Tract, No. 81

**Published:** 1862-07

**Authors:** 


HEALTH TRACT, No. 81.

---

### MUSIC

REFINES the taste, purifies the heart, and elevates our nature. It does more: it soothes in sorrow, tranquillizes in passion, and wears away the irritabilities of life. It intensifies love, it fires patriotism, and makes the altar of our devotion burn with a purer, holier flame. Not only man, but the brutes themselves have been restrained and charmed by the bewitching power which it possesses. And in the still twilight hour, when sweet, sad memories go back upon the distant past, and hover lovingly about the places where we played and the persons whom we loved, but now gone, in their youth and beauty and purity, to return no more, who does not know that the soul drinks more deeply in of the saddening sweetness when it breaks out in the soft, low notes of song, or the fingers instinctively sweep through diapasons absolutely ravishing? And when tedious disease has dampened the fires of life, has removed its gilding and written "vanity" on all things earthly; when wealth and fame and worldly honor are felt to be nothing; when the aims and ambitions and aspirations which were wont to rouse up all the energies of nature toward their accomplishment fail of their accustomed power, music renders the burden of sickness light, and makes us all oblivious of pain and suffering. For these reasons, that parent has largely neglected a religious duty, has been strangely forgetful of one of the highest of all obligations, who fails to afford his children, while yet young, all the facilities in his power for fostering and cultivating whatever taste for music they possess, whether vocal or instrumental; for in after-life, and through all its vicissitudes, those who practise it, in the love of it, when young, will find in its exercise a happy escapade in seasons of boisterous mirth, and thus increase the joy; in times of depondency, its expression will give encouragement; when difficulties oppose, it will inspire strength to overcome them, and when clouds of trouble gather around and above, hedging up the future, shutting out the blue sky of life, music can penetrate even Egyptian darkness, and let in upon the almost broken heart the sunshine of hope, of gladness, and of joy.

It is because of this view of the health-giving, happifying, and refining influences of music, that in the progress of a high civilization its cultivation has become a profession, not only among those who give utterance to it in vocal symphonies, "almost divine," but men of genius and mechanical talent, not willing to be laggards in their department, have brought all their energies to bear in the improvement of every form of instrumental music, and, more than in any other direction, on the piano-forte, which, as all believed, had been brought to a point of perfection in tone and chord and symphony which could scarcely be improved upon. But, lo! within a month, "Letters Patent" have been taken out by a gentleman of this city, claiming an improvement, of which the Hon. Erastus Brooks, of the *New-York Daily Express*, says, under date of Wednesday, May 28th, 1862:

"Horatio Worcester, one of the most successful city piano manufacturers, has obtained Letters Patent for an improvement in the piano-forte, consisting of a hinged-plate made in two pieces, the stationary part being fastened to the instrument in the usual manner. The piece to which the strings are hitched is constructed separately, and has a link or opening in the base end, by which means it is suspended from an abutment on the fixed portion of the plate. Thus is formed a hinge or coupling on which the detached piece moves freely in connection with the strings while they are in operation, the effect of which is to communicate increased vibratory power throughout the whole extent of the sounding-board under the plate. This new principle has been successfully tested on several instruments. The increased vibration produces a singing quality of tone unusually powerful and agreeable. The inventor produces instruments of the rarest excellence, as can be seen and heard upon examination."

In reference to the same improvement, the *New-York Commercial Advertiser*, now in the sixty-eighth year of an honorable age, and of which all its *confrères* speak uniformly with consideration and respect, adds:

"SOMETHING NEW IN PIANOS.—Mr. H. Worcester, the well-known manufacturer of pianos, has just taken out a patent for a valuable improvement in pianos.

"'This improvement consists in the use of a hinged-plate, which gives to the sounding-board a freedom similar to that found in the violin. The plate is made in two pieces, the stationary part being fastened to the instrument in the usual manner. The piece to which the strings are hitched is constructed separately, and has a link or opening in the base end, by which means it is suspended from an abutment on the fixed portion of the plate. Thus is formed a hinge or coupling upon which the detached piece moves freely in connection with the strings, while they are in operation, the effect of which is to communicate increased vibratory power throughout the whole extent of the sounding-board under the plate.

"'The increased vibration obtained produces a singing quality of tone unusually powerful and agreeable, while for general volume, durability, evenness, and richness of tone, the inventor claims that these excel any piano-fortes that he has heretofore produced.'"

In other words, sweet as were the tones of the piano before, Worcester's improvement makes it

"A" (*lengthened*) "sweetness, long drawn out,"

reminding us of the man who, after draining his glass of the very last drop, exclaimed, with a most distressful sigh, "I wish——" and there stopped, and began to wring his hands.

"What do wish?" said a bystander, in a very impatient tone.

"I wish my neck was a mile longer; it was so good in going down."

